# A Unified Framework for Head Pose, Age and Gender Classification through End-to-End Face Segmentation

**DOI:** 10.3390/e21070647

**Published:** 2019-06-30

**Authors:** Khalil Khan, Muhammad Attique, Ikram Syed, Ghulam Sarwar, Muhammad Abeer Irfan, Rehan Ullah Khan

**Affiliations:** 1 Department of Electrical Engineering, University of Azad Jammu and Kashmir, Muzafarabbad 13100, Pakistan; 2 Department of Software Engineering, Sejong University, Seoul 05006, Korea; 3 Department of Software Engineering, University of Azad Jammu and Kashmir, Muzafarabbad 13100, Pakistan; 4 Dipartimento di Elettronica e Telecomunicazioni (DET), Politecnico di Torino, 10156 Torino, Italy; 5 IT Department, College of Computer, Qassim University, Al-Mulida 51431, Saudi Arabia

**Keywords:** face analysis, face segmentation, head pose estimation, age classification, gender classification

## Abstract

Accurate face segmentation strongly benefits the human face image analysis problem. In this paper we propose a unified framework for face image analysis through end-to-end semantic face segmentation. The proposed framework contains a set of stack components for face understanding, which includes head pose estimation, age classification, and gender recognition. A manually labeled face data-set is used for training the Conditional Random Fields (CRFs) based segmentation model. A multi-class face segmentation framework developed through CRFs segments a facial image into six parts. The probabilistic classification strategy is used, and probability maps are generated for each class. The probability maps are used as features descriptors and a Random Decision Forest (RDF) classifier is modeled for each task (head pose, age, and gender). We assess the performance of the proposed framework on several data-sets and report better results as compared to the previously reported results.

## 1. Introduction

The problem of human face image analysis is a fundamental and challenging task in computer vision. It plays a key role in various real world applications such as surveillance, animation and human computer interaction. However, it is still a challenging task due to changes in facial appearance, visual angle, complicated facial expressions and the background. In particular, in the un-constrained conditions it has much more complications.

Each of these face analysis tasks (head pose, age and gender recognition) are approached as individual research problem through various sets of techniques [1,2,3,4,5,6,7,8]. We argue that all these tasks are *very closely* related and essentially can help each other if a prior efficiently segmented face image is given as input. It is also confirmed by psychology literature that face parts such as nose, hair, and mouth helps human visual system in face identity recognition [9,10]. Therefore, performance of all related applications can be improved if a well segmented face image is provided as input to the framework.

The facial attribute information such as head pose estimation, age classification, and gender recognition is already being predicted using facial landmarks information [4,11]. However, the performance of head pose and any other applications in such cases heavily depends on accurate localization of these landmarks [5,7,12]. Locating these face landmarks is itself a *big challenge*. These points localization are greatly affected in certain cases such as occlusion, face rotation and if the quality of the image is very low. Similarly, in far-field imagery conditions, these landmarks extraction are not only difficult but some-times impossible. Lighting conditions and complicated facial expressions also make the localization part challenging. Due to all problems mentioned above, we approach the face analysis task in a complete different way.

In this paper we introduce a unified framework, which addresses all the three face analysis tasks (head pose, age, and gender recognition) through a prior multi-class face segmentation model that was developed through CRFs. We named the newly proposed multitask framework HAG-MSF-CRFs. It is a jointly estimation probability task that tackles it using a very powerful random forest algorithm. Specifically, the proposed framework can be formulated as;

(1) $$\left(h,a,g\right)=\underset{h,a,g}{arg max}p\left(h,a,g|I,B\right)$$

where head pose, age, and gender recognition are represented by *h*, *a* and *g* respectively. Similarly, in Equation (1), *I* is the input face image and *B* is the bounding box which is provided by the face detector.

In our previous work we already tackle the problem of multi-class semantic face segmentation (MSF) [13] and its application to head pose estimation [14,15] (MSF-HPE) and gender classification [16]. In most of the previous works, face segmentation is considered as three or some-times four classes face segmentation task. In the MSF, face segmentation is extended to six classes (eyes, nose, mouth, skin, back and hair). However, we were facing some major problems in previously proposed MSF. Firstly, the computation cost of MSF is quite high, as MSF provides a class label to each and every pixel in an image, which ultimately takes a long time. A super-pixel based model is used instead which reduces the processing cost. Secondly, the MSF does not consider any conditional hierarchy between different face parts. For example, it is not possible for the eye region to be near to the mouth region and vice versa. A CRFs based model is introduced in this paper, which couples all labels in a face image in a scaled hierarchy. Going from MSF to the newly proposed MSF-CRFs improves the performance of the segmentation part.

Our proposed multi-task framework is comparable to another approach known as the influence model (IM). This model was first introduced by researchers in the MIT media laboratory [17,18]. The IM estimates how the state of one actor affects another in the system. Our proposed model is somehow similar to the model proposed in [17,18]. In such cases, an outcome in one entity in a system causes outcome in another entity in the same system. In simple words, if one domino is flipped, the next domino will fall automatically and vice versa. In IM it is necessary to know how certain dominoes interact with each other and how one is influenced by another. If the initial state of the dominoes is known with relative location to another, then the outcome of the system is predicted with more accuracy. When the system network structure is already known, the IM enables researchers to infer interaction; however, information about signals from different observations are needed.

To summarize, contributions of the paper are three fold:

- We propose a new multi-class face segmentation algorithm MSF-CRFs. The MSF-CRFs model uses the idea of CRFs between various face parts.
- We develop a new multi-tasks face analysis algorithm HAG-MSF-CRFs. The HAG-MSF-CRFs tackles all the three tasks, which include head pose, age, and gender recognition in a single framework.
- Detailed experiments are conducted on state-of-the-arts (SOA) data-sets, and better results are reported comparatively.

The structure of the remaining paper is as follows: Section 2 describes related works for all the three cases i.e., head pose, age, and gender recognition. Several data-sets are use to evaluate the framework. Details about these databases is given in Section 3. The segmentation model MSF-CRFs is presented in Section 4, whereas the proposed algorithm for face analysis (HAG-MSF-CRFs) is discussed in Section 5. All obtained results are discussed and compared with SOA in Section 6. The paper is summarized with some future directions in Section 7.

## 2. Related Work

Our newly proposed model is closely related to IM based built systems. The IM framework is already used in the automatic recognition tasks of social and task-oriented functional roles in group-meetings [17,18]. The classification of social functional roles has been improved as compared to Hidden Markov Models (HMM) and support vector machine (SVM) [18] through IM. The two versions proposed in [18] outperform both HMM and SVM based results in the social functional role problems. The IM methods showed excellent performance, particularly in less populated classes. Media segmentation is performed with IM in cases particularly having rich information [19,20,21]. The keywords information are exploited in [22] to identify journalists, anchors, and guest speaker if any in a radio program. The maximum entropy algorithm is used for getting the classification accuracy. The IM based algorithms are applied to many audio and visual recognition tasks, for details, more papers can be explored in [23,24,25,26,27,28].

Before describing the proposed framework, we briefly review related methods for head pose, age, and gender classification. A rich literature and history is already present about all these three topics. However, in this section of the paper we provide a cursory overview of how these tasks were previously approached by researchers.

### 2.1. Head Pose Estimation

Pose of an image can be classified into three broad categories; yaw, pitch, and roll. The yaw angles represents the horizontal orientation and the pitch vertical orientation of a face image. The image plane is represented by the roll angles. We evaluated our proposed algorithm for head pose estimation on four data-sets, which included Pointing’04 [29], Annotated Facial Landmarks in the Wild (AFLW) [30], Boston University (BU) [31], and ICT-3DHPE [32] data-sets.

Two types of information were previously used to approach the head pose estimation i.e., facial landmarks and face image appearance. In the former case, a POSIT algorithm [9] is used to find correspondence between pints in 2D shapes and points in 3D models. In the latter case, various image appearance features such as SIFT, LBP, HOG etc. are exploited for head pose estimation. Discriminative learning models such as Random Forest and Support Vector Machine (SVM) are trained and tested using the extracted features [4,10]. A more detailed survey on head pose estimation can be explored in [5].

### 2.2. Age Classification

Age classification is a well-researched topic in computer vision society. Previously, age estimation was studied as a classification or regression problem. In the first case, age is associated with a specific range or age group. In the second case, the exact age of a face image is estimated. Recently a survey paper was reported on age estimation in [33]. All data-sets used for age estimation were discussed and a detailed overview was presented about the algorithms proposed thus far. A detailed investigation of age classification between specific ranges or age groups was presented in [34]. Similarly, another algorithm is introduced to classify age from facial images in [35]. Initially, the appearance of face wrinkles is detected and then age categorization is performed based on the extracted wrinkles. The previous idea [35] was further extended in [36] by first localizing the facial features. The modeling of craniofacial growth was performed through psychophysical and anthropometric evidences in [36]. The main drawback of this approach was: accurate localization of facial features is needed in any case.

A subspace method called AGing PatErn subspace is introduced in [37,38]. In these algorithms, aging features from face images were extracted and an adjusted robust regressor was trained to categorize face ages. These methods showed excellent performance compared to SOA methods. However, two serious weaknesses are faced by these algorithms. The input images must be frontal, and the face images must be well-aligned. The approaches proposed in these algorithms are suited for databases collected in indoor environmental conditions. Practical applications of these methods in the un-constrained conditions is almost impossible.

A cost-sensitive hyper-planes ranking method is introduced in [39]. The algorithm proposed in [39] is a multi-stage learning method which is also known as ‘a grouping estimation fusion’ (DEF) method in the literature. Similarly, a novel features selection method was proposed in [40]. In a nutshell, all these previously mentioned methods showed good performances in indoor lab conditions, but failed when exposed to the real-world conditions.

Recently introduced Deep Convolutional Networks (CNNs) showed excellent performance for different visual recognition problems. A hybrid system for age and gender classification is proposed in [41]. CNNs are used to extract features from the face images, whereas an extreme learning machine (ELM) is used as a classification tool. The authors of the paper named their proposed method as CNNs-ELM. The system is evaluated on two data-sets, MORPH-II [42] and Adience [43]. To the best of our knowledge, this is the best algorithm performing on a joint problem of gender and age recognition thus far. A weakness reported by the authors of the paper is: miss-classification occurs when the system is exposed to younger faces.

### 2.3. Gender Classification

A detailed investigation about gender recognition was conducted by Makinen and Raisamo [44]. The early researchers who worked on gender recognition used neural network [45]. An SVM classifier was used by Moghaddam and Yang [46]. Similarly, an Adaboost classifier was adapted by Baluja and Rowley [47]. In all these methods image was used as one dimensional feature vector and certain features are extracted from it. A joint framework of age and gender recognition was proposed by Toews and Arbel [48]. The model proposed by the authors is a view-point invariant appearance model which is robust to local scale rotations.

Gender classification analysis based on human gait and linear discriminant algorithms was provided by Yu et a. [49]. A new benchmark to study age and gender classification was suggested in [43]. Through the available data, a classification pipeline is presented by the authors of the paper. Khan et al. [50] proposed a semantic pyramid, dealing both gender and action recognition. Annotation for face and upper body was not needed in the proposed method. First part of the name was used as a feature and a modeling mechanism of the name part and face images was performed in the next stage in a method proposed in [51]. Higher accuracy was reported with proposed method as compared to SOA. Recently, a generic algorithm to estimate gender, race, and age in a single framework is proposed in [52].

All the above-mentioned approaches made lots of progress and contribution towards gender recognition. However, most of these methods were aimed either at non-automated estimation methods or only worked well in very constrained imaging environments.

## 3. Databases

In this paper we use six different face databases to perform the three tasks i.e., head pose, age and gender classification. For head pose estimation we use Pointing’04, AFLW, BU, and ICT-3DHPE data-sets. For age classification we use Adience and FERET [53] data-sets. For gender recognition we perform tests with Adience database only.

### 3.1. Head Pose Estimation

**Figure 1:**
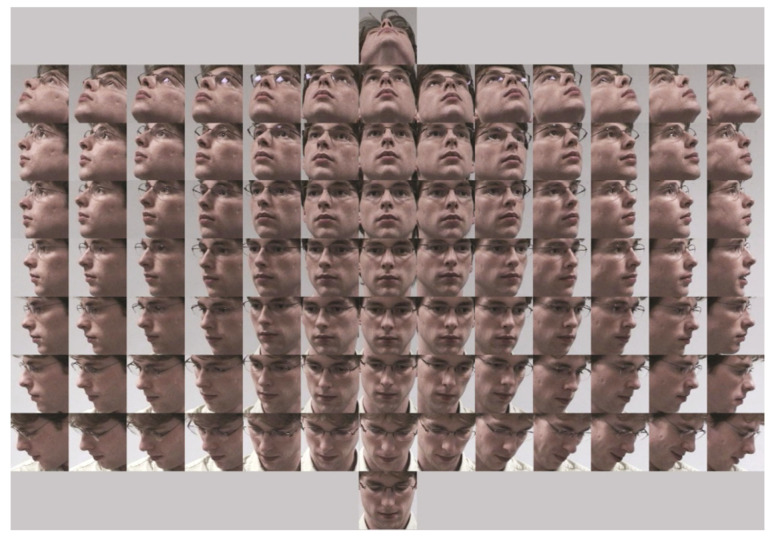
Pointing’04 database images of a single subject in all 93 poses.

- **Pointing’04 database:** The Pointing’04 database is a manually annotated face database. Even though it is a comparatively old head pose data-set, it is still used for research purposes [54,55,56] due to its challenging nature and large variety with consecutive poses. All the images in the Pointing’04 database are low resolution images captured in low lighting conditions. The Pointing’04 contains 15 sets of face images. Each set is further divided into 2 sets having 93 images for each candidate at various orientations. The age of each subject in the database is kept between the range 20–40 years. To add more complexity to the database images, five subjects were included with facial hair and seven were wearing glasses. The pan and tilt angle determined the head pose of a subject. Each subject in the database acquisition was asked to look into 93 markers marked on the wall. Each marker represented a specific pose. The given face localization in Pointing’04 may not be accurate due to manual labeling. A sample of the images of a single candidate at 93 different locations is shown in Figure 1. For yaw, the head orientation varied between −90${}^{{}^{\circ}}$ to +90${}^{{}^{\circ}}$ with a step size of 15${}^{{}^{\circ}}$ between two adjacent poses. For pitch, the positive values corresponded to the top poses and negative to the bottom poses. The difference between two consecutive poses in the pitch is 30${}^{{}^{\circ}}$.
- **AFLW Data-set:** Images in AFLW exhibited variations in facial expression, lighting conditions, face appearance, and some other environmental factors. All these images were obtained from the internet. The AFLW contained both the frontal and non-frontal images. The frontal images had six facial expressions. More difficulties were added to the images in the form of certain facial accessories. The images were collected from 9 different lighting conditions. In short, AFLW is a very challenging data-set, since the data-set is collected in the real world with un-constrained conditions.
- **BU Data-set:** The BU data-set has two image sequences, i.e., images collected in uniform lighting conditions and images exposed to rather complex scenarios by changing the lighting conditions. We used RGB images only for the experiments. We considered all the three rotations, which included pitch, roll and yaw. A total of 5 subjects participated in the image acquisitions process. A magnetic tracker was attached to each subject’s head to obtained the ground truth images.
- **ICT-3DHPE:** A Kinect sensor was used to collect the ICT-3DHPE images. This data-set contains both the depth and RGB images. However, we only used the RGB images in our work. Six male and four females participated in the image collection process. The ground truth images were more accurate in this case as well (like BU data-set), because a magnetic tracker was attached to each participant’s head. It must be noted that the ground truth images creation method for Pointing’04 and AFLW is a type of manual labeling method. The chances of error exists while providing labels to the ground truth data.

### 3.2. Age and Gender Classification Data-Sets

- **Adience Benchmark:** It is a recently released un-constrained image database which is used both for age and gender recognition. All these images were created from smart phone devices. These images included variations such as pose, lighting, appearance, noise, and more—meaning the data-set has all conditions of un-constrained image database. The total number of images in Adience are 26,580, whereas the total number of participants are 2284. The exact age of each candidate is not specified, and each subject is assigned to 8 different age groups i.e., [0,2], [4,6], [8,13], [15,20], [25,32], [38,43], [48,53], [60,+]. The data-set can be obtained from the Open University of Israel (computer vision lab).
- **LFW data-set:** The LFW database consists of 13,233 images for 5,749 subjects. The data-set was collected in un-constrained conditions. All these face images were collected from the web. It is an imbalanced database, because the number of male candidates are 10,256 whereas female images are 2977.
- **FERET data-set:** This is also an old data-set that is widely used to develop and evaluate various facial recognition methods. The database was collected in controlled indoor conditions with gender information for each subject. The data-set is composed of 14,126 images whereas the total number of participants were 1,199. We used the colored version from the FERET database. Some variations of facial expressions, lighting conditions and face pose were kept while image acquisition—made the database a rather challenging one. The database consists of both frontal and non-frontal images. We applied our algorithm to both set of images (between −45${}^{{}^{\circ}}$ and +45${}^{{}^{\circ}}$).

## 4. Proposed MSF-CRFs

**Figure 2:**
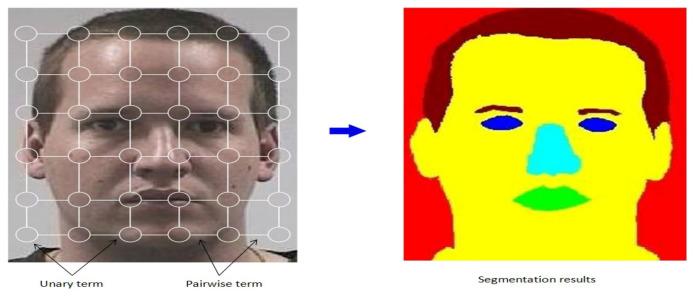
The MSF-CRFs graphical model. The input face image in grid cell represents a random variable. The unary potentials are represented by the white circles and the pairwise potential by solid white lines.

The overview of the MSF-CRFs model for semantic face segmentation is shown in Figure 2. The labeling problem is modeled efficiently with the proposed MSF-CRFs, which combines the output from the built classifier with image location information. This modeling process helps in maximizing a posteriori. The unary potential models each pixel belonging to each class and the pairwise potential models the relationship between two pixels.

As face parts are not localized in most of the images, a face localization algorithm is applied in start. In the literature there are many good methods for face detection, so we use a CNNs based face detector [57]. After localizing the face parts, all face images are re-scaled to a fixed size with a height 256 pixels and the width is adjusted accordingly to keep the original image ratio.

The proposed MSF-CRFs model encodes segmentation probability with features of an image. Initially an image is segmented into super-pixels. The segmentation is represented by ***Z*** and this can be represented as ***Z*** = $z_{1},z_{2},\ldots ,z_{n}$, where n is the total number of super-pixels in the input image. $z_{i}$ can take the value of any of the six face parts (nose, eyes, mouth, hair, back and skin). For super-pixel segmentation we use SEEDs [58] algorithm.

We also need to develop some conventions about node and edge features. We represent the node features by $Z_{m}$ and edge features by $Z_{e}$. We develop a log linear CRFs model which can be written as:

(2) $$\psi \left(s_{i}=q,z_{i}^{m}\right)=\sum_{f=1}^{F_{m}}{\left(X_{q}^{m}\right)}_{f}{\left(z_{i}^{m}\right)}_{f}$$

(3) $$\psi \left(s_{i}=q_{1},s_{j}=q_{2},=z_{i,j}^{e}\right)=\sum_{f=1}^{F_{e}}{\left(X_{q_{1},q_{2}}^{e}\right)}_{f}{\left(z_{i,j}^{e}\right)}_{f}$$

In Equations (2) and (3), super-pixel features are represented by F${}_{m}$ whereas Z${}_{i}^{m}$ represents a vector having length F${}_{m}$. The neighboring super-pixels features are represented by F${}_{e}$. The final resultant feature vector developed is Z${}_{i,j}^{e}$. Similarly, each node and edge weight are adjusted with $X^{m}$ and $X^{e}$ respectively. A pair of classification labels in the above Equations is represented by *q${}_{1}$, q${}_{2}$*. In the proposed MSF-CRFs model we use symmetric edge potential.

The probability of segmentation conditional on *Z* can be represented as:

(4) $$P\left(s|z\right)=\frac{exp(-\sum_{i=1}^{m}\psi \left(s_{i},z_{i}^{m}\right)-\sum_{i,j}\psi \left(s_{i},s_{j},z_{i,j}^{e}\right))}{N(Z)}$$

N(Z) represents the partition function in Equation (4). This function acts as a normalization factor for the distribution. We use Bethe Approximation [55] for the partition function in the MSF-CRFs model. Similarly, for marginal approximation we use a loopy belief propagation algorithm. For CRFs optimization, we use the algorithm as in L-BFGS [59]. For weight regulations we also added the Gaussian to the model.

To assess the accuracy of the segmentation estimates, we apply an L1 error to each segmentation estimate. We also penalize each super-pixel as per the difference between the correct label prediction probability and a value 1.0. For example, if a super-pixel has a probability value of 0.7 for being skin (and skin is also the ground truth label of the super-pixel), a penalty value of 0.3 will be incurred as a result.

We compute three types of features for the node listed as; position, HSV color and shape related information (HOG).

For spatial information an 8 × 8 grid is considered, and then the relative location of the central pixel is extracted. This location is defined as:

(5) $$f_{loc}=\left[x/W,y/H\right]\in R^{2}$$

where *W* represents the width and *H* height of the input face image.

For color features, the information from HSV histogram is extracted. The three values (hue, saturation, and variance) are encoded in a single vector constituting a unique feature vector for color information. The dimension of each patch for HSV is kept as $D_{HSV}$= 16 × 16, whereas the number of bins are set 32. The resulting feature vector for the color information with these values will be $F_{HSV}^{16}\in R^{48}$.

For shape information we use HOG. We keep the dimension of the patch for HOG as $D_{HOG}$= 64 × 64, which results a feature vector $F_{HOG}^{64\times 64}\in R^{1764}$.

All the three features are concatenated with each other to form a single vector.

## 5. Proposed HAG-MSF-CRFs

**Figure 3:**
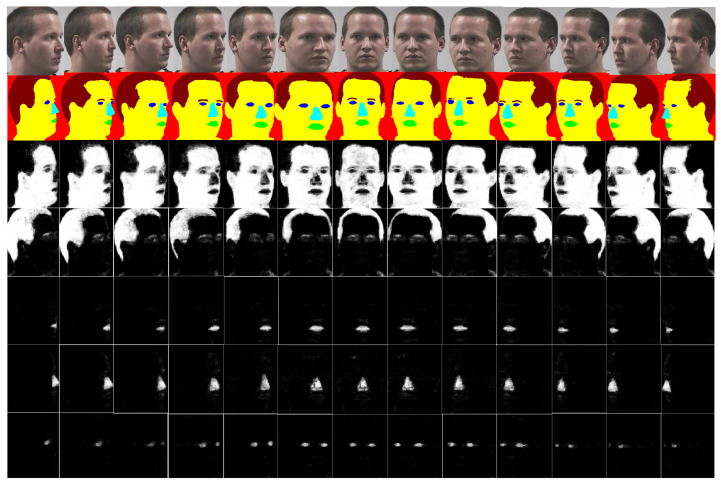
Probability maps of a single subject from Pointing’04. Poses vary from −90${}^{{}^{\circ}}$ to +90${}^{{}^{\circ}}$ with a step of 15${}^{{}^{\circ}}$ in the horizontal orientation. Row wise order of the images is as: 1—original images, 2—ground truth images, 3—probability maps for skin, 4—probability maps for hair, 5—probability maps for mouth, 6—probability maps for nose, and 7—probability maps for eyes.

Our proposed algorithm is summarized in Algorithm 1. Initially a segmentation model is developed through the CRFs. For face segmentation, the built model MSF-CRFs outputs the most likely class for each super-pixel. The same label is then assigned to each pixel within the super-pixel. For the classification of head pose, age and gender we use the probability maps created during segmentation of each class. Probability maps generated for each class are represented as: ***P*****${}_{nose}$**, ***P*****${}_{back}$**, ***P*****${}_{eyes}$**, ***P*****${}_{skin}$**, ***P*****${}_{mouth}$**, and ***P*****${}_{hair}$**. Figure 3 show some images from Pointing’04 data-set and their probability maps. In the gray-scale images in Figure 3, higher intensity represents higher probability of prediction for a particular class and *vice versa*. For each task (head pose, age, and gender) we train an RDF classifier with a feature vector of the corresponding probability maps. The probability maps are used as feature descriptor.
**Algorithm 1** proposed HAG-MSF-CRFs algorithm**Input:*****M***${}_{train}$ = {(*I${}_{n}$*,*T${}_{n}$*)}${}_{n=1}^{m}$, ***M*****${}_{test}$**.where ***M*****${}_{train}$** is the data used for training model A, ***M*****${}_{test}$** is the testing data, *I* is the input training image and *T(i,j)*∈ {1,2,3,4,5,6} is the ground truth data.**a: Face segmentation part:**Step a.1: Training a segmentation model A through training data (training images and labels)Step a.2: Finding the center of each super-pixel, extracting patches and passing to the model AStep a.3: Using the probabilistic classification method and creating probability maps for each class, represented as:  *p*${}_{skin}$, *p*${}_{mouth}$, *p*${}_{eyes}$, *p*${}_{nose}$, *p*${}_{hair}$, and *p*${}_{back}$**b. Head pose, age and gender classification part:**  **if** head pose estimation:  f=*p*${}_{skin}$ + *p*${}_{mouth}$ + *p*${}_{eyes}$ + *p*${}_{nose}$ + *p*${}_{hair}$  **Else if** age classification:  f=*p*${}_{skin}$ + *p*${}_{mouth}$ + *p*${}_{eyes}$ + *p*${}_{nose}$ + *p*${}_{hair}$  **Else if** gender recognition:  f=*p*${}_{skin}$ + *p*${}_{eyes}$ + *p*${}_{nose}$ + *p*${}_{hair}$where f is the feature vector.c. Training an RDF classifier for each case (head pose, age and gender)**Output:** estimated pose, age class and gender.

### 5.1. Head Pose Estimation

We manually labeled 10 images from each pose of each data-set. The manually labeled images are used to build an MSF-CRFs model as discussed previously. For all images of every data-set, the probability maps are generated. When a test image is given as input, the MSF-CRFs model creates the probability maps for all classes and all images.

To understand which facial parts help in head pose estimation we conducted a large number of experiments. We use probability maps for the eyes, nose, mouth, skin, and hair. Probability maps in the form of feature descriptors are concatenated to train and test an RDF classifier. We use 10-fold cross validation experiments in our work. Those 10 images, which were previously used to create an MSF-CRFs model were not included in the 10-fold cross validation experiments. The probability maps of a single subject from Pointing’04 data-set are shown in Figure 3. From the Figure 3, it is clear that variation occurs as the pose changes from one position to another. For example taking the skin class (third row), forehead is more exposed to the camera in frontal images. As a result, probability map for brighter part is more concentrated to the center part. Similarly, on extreme left and right profile images, high intensity values are occupied on smaller area. We encoded this information for all classes in the form of feature descriptors and developed a new head pose estimation algorithm.

### 5.2. Age Classification

In age classification a face image is assigned to one of the specific age range. From each age group of each data-set, 10 images are manually labeled. The manually labeled images are used to build an MSF-CRFs model. The test face images are passed to the MSF-CRFs model to produce segmentation results and probability maps.

We noted during the experiments that each face part has a contribution towards age classification. Probability maps for each face part differ from one age group to another. Therefore, for age classification we use information about all five face classes, i.e., skin, mouth, hair, and eyes. The probability maps generated are used to train and test an RDF classifier. As in case of head pose, 10-fold cross validation experiments are performed here as well. Manually labeled images which were previously used to create MSF-CRFs model were not included in the 10-fold cross validation experiments.

### 5.3. Gender Recognition

For gender classification, we manually label 30 images for each gender and each data-set. These total 60 images are used to build an MSF-CRFs model for the gender test. A number of qualitative and quantitative experiments are conducted to know which face parts help in gender recognition. After these experiments we train an RDF classifier through probability maps of four classes namely; nose, hair, eyes, and skin.

We perform a detailed study from computer vision and human anatomy literature to know which face parts make a face more feminine or masculine. In the following paragraphs we summarize why we use four classes (skin, nose, hair, and eyes) for gender recognition.

**Figure 4:**
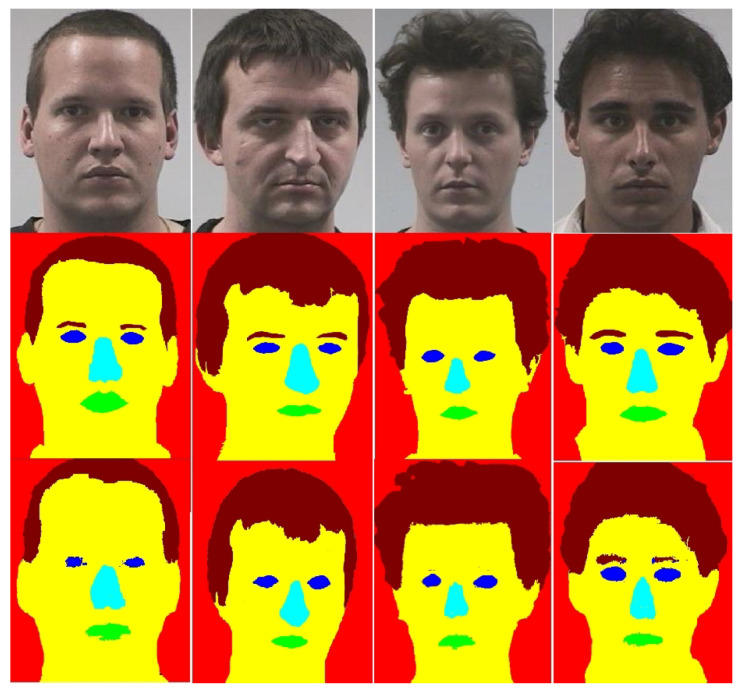
Face segmentation results with MSF-CRFs for frontal images on Pointing’04. Images in rows are in order as: row 1—original images, row 2—manually labeled images, row 3—segmentation results produced by MSF-CRFs.

**Figure 5:**
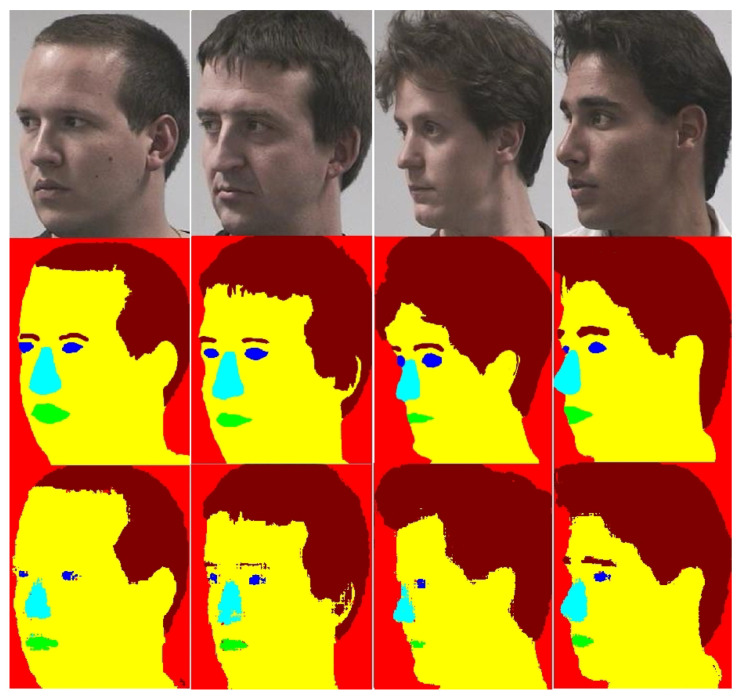
Face segmentation results with MSF-CRFs for profile images (+60${}^{{}^{\circ}}$) on Pointing’04. Images in rows are in order as: row 1—original images, row 2—manually labeled images, row 3—segmentation results produced by MSF-CRFs.

- Usually male forehead is larger compared to female—as the hair line in male lags behind. In male hairline is completely missing in some cases (baldness). This results a larger forehead in male as compared to female. Consequently, brighter part of probability map for the skin is on larger area in case of male.
- Female eyelashes are larger and curly type. Our MSF-CRFs part mis-classified these eyelashes with hairs in females in most of the cases. Even this mis-classification reduces the pixel labeling accuracy of the segmentation part. However, this helps the gender differentiation. In the case of male, pixel labeling accuracy noted was 79%, resulting better segmentation with brighter probability map. For female the labeling accuracy reduced to 69%, which results a comparatively dimmer probability map.
- A female nose is comparatively smaller with less bridge. On the other hand, male nose is larger and also comparatively longer. A reason reported in the literature for this fact is: as compared to female, the male body is bigger which requires larger lungs and enough passage of air supply towards lungs. Consequently, the male nostrils are larger than female.
- Hairstyle has a very complicated geometry that varies from subject to subject. Our proposed MSF-CRFs reports a pixel labeling accuracy of 97.23%. From the segmentation results (please see Figure 4 and Figure 5), it is clear how efficiently boundary line for hair is detected by our MSF-CRFs model. We encode this information in the form of probability maps and used it in the gender recognition part.
- Sometimes, even eyebrows also help in gender recognition. Male eyebrows are mis-managed and larger, whereas female eyebrows are thinner and curl at the end. In our face segmentation model, we use the same label for eyebrow as hair.
- Literature reports that the mouth must help male and female differentiation. Female lips are clear and visible, whereas in most of the cases upper lip is somehow missing in male images. Unfortunately, we noted no improvement in gender recognition performance with inclusion of the mouth class. Therefore, we did not include mouth class for gender recognition algorithm.

Thus, probability maps for skin, nose, hair, and eyes are concatenated with each other to form a single feature vector. We perform 10-fold cross validation experiments here as well. However, we excluded 60 images which were previously used for training part from each database tests.

## 6. Results and Discussion

### 6.1. Face Segmentation Results

To the best of our knowledge, previously proposed MSF is the first work that considered all six face parts in face segmentation. The main problem with MSF is its computational cost. To remove this deficiency, we used a super-pixel based segmentation in the current model (MSF-CRFs). The processing time of segmentation was improved four times with the MSF-CRFs as compared to the MSF. For example, an image with a 256 × 240 pi size took 1.2 min in the MSF model. The same image was segmented with MSF-CRFs in just 18 seconds.

An image is segmented into super-pixels initially. Super-pixel segmentation reduces processing time of segmentation as the number of pixels to be labeled are reduced immensely. In the proposed method we used SEEDs [58] algorithm for super-pixel segmentation. We prefer SEEDS over SLIC and other methods as the speed of the SEEDS is much better than other methods used in SOA [58]. Moreover, SEEDS has much better super-pixel segmentation as reported in standard error metrics.

Face segmentation results for frontal images are much better than profile images. For different super-pixel parameters setting we performed experiments. We noticed better segmentation results with 900 super-pixels. The exact number of super-pixels were less than 900 due to certain segmentation restrictions. The number of super-pixels obtained during the experiments depended on the block levels used and the image size. The super-pixel segmentation was better when the block levels were higher. We used the number of block levels 3, and histogram bins 5. For better accuracy iteration accuracy was kept twice.

Few images from Poinint’04 dataset are shown in Figure 4 and Figure 5. Figure 4 shows some good segmentation results. In Figure 4 and Figure 5, the first row shows the original images, row 2 shows manually labeled images and row 3 shows images segmented with the MSF-CRFs. The frontal images are segmented in Figure 4, whereas the same images rotated at +60${}^{{}^{\circ}}$ are shown in Figure 5. From these Figs. it is clear that pixel labeling accuracy for frontal images is much better than profile images. It can be noted that as the pose moves to the left or right, labeling accuracy dropped particularly for smaller classes (eyes, nose, and mouth). For extreme profile poses (+90${}^{{}^{\circ}}$ and −90${}^{{}^{\circ}}$) these smaller classes in some images were completely missing.

Performance of the segmentation part highly depends on the quality of the images as well. For example, in the case of AFLW data-set, the images were collected from the internet which included very low quality images. Therefore, poor segmentation results were noticed, ultimately leed to the poor performance of head pose and gender recognition.

### 6.2. Head Pose Estimation

We used two evaluation methods for head pose estimation. The first one is a regression measure i.e., mean absolute error (MAE). MAE is the absolute error between the estimated and ground truth pose. The second one is a classification measure i.e., pose estimation accuracy (PEA). PEA estimates how a particular pose is predicted by a model.

**Pointing’04 data-set:** The results obtained with HAG-MSF-CRFs on the Pointing’04 data-set and its comparison with SOA for both yaw and pitch angles is shown in Table 1. From the Table 1, it is clear that we achieved better results as compared to previously reported results for both the MAEs and PEAs. All possible combination of the six face classes were tried in the experiments. The best results for yaw (average MAE = 2.32${}^{{}^{\circ}}$ and average PEA = 87%) and pitch (average MAE =1.18${}^{{}^{\circ}}$ and average PEA = 95%) were obtained with five classes i.e., ‘nose’, ‘mouth’ ‘skin’, ‘hair’, and ‘eyes’. It must be noted that some of the previous methods mentioned in Table 1 may have used a differential experimental setup. For example, 5-fold cross validation experiments were performed in the MLD. We performed our experiments with 10-fold cross validation protocol. Corresponding papers can be explored for the experimental setup and more details for each case.

**Figure 6:**
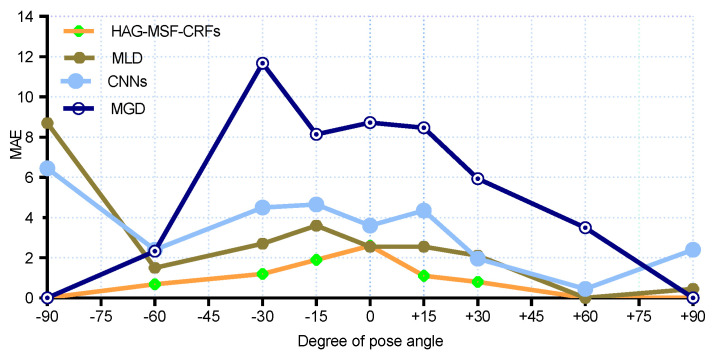
MAE comparison with SOA on Pointing’04 (pitch).

**Figure 7:**
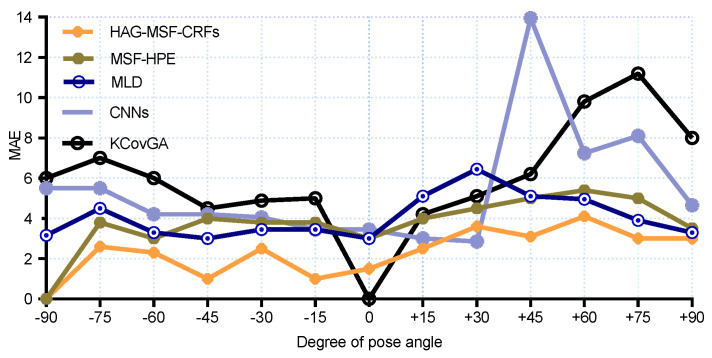
MAE comparison with SOA on Pointing’04 (yaw).

**Figure 8:**
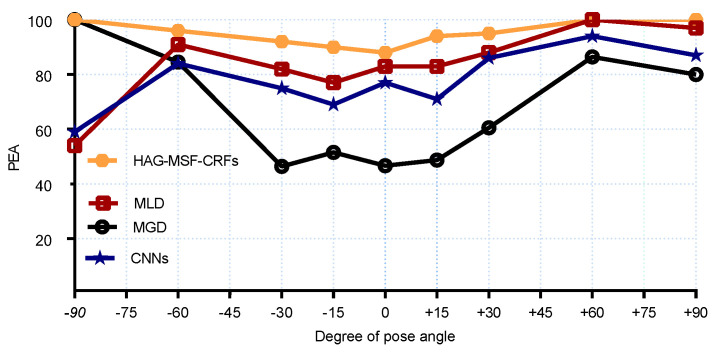
PEA comparison with SOA on Pointing’04 (pitch).

**Figure 9:**
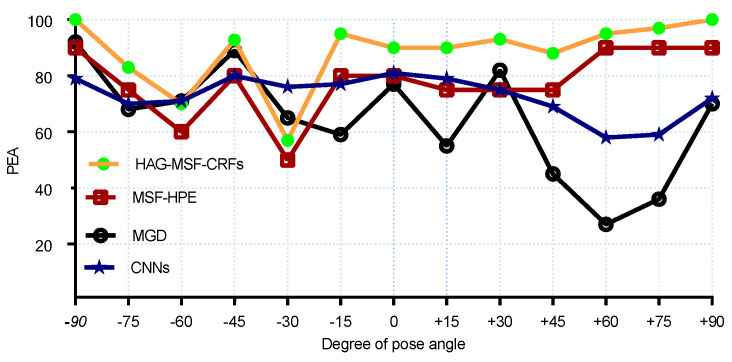
PEA comparison with SOA on Pointing’04 (yaw).

For a more clear comparison with SOA methods, we also reported the results for each pose both for the MAEs and PEAs. The MAEs results are compared in Figure 6 and Figure 7 for pitch and yaw angles respectively. We had the best results for MAE for all yaw poses (except, 0${}^{{}^{\circ}}$ and +30${}^{{}^{\circ}}$). Similarly, Figure 8 and Figure 9 shows the PEAs results obtained with proposed method and its comparison with SOA for each discrete pose. From the Figure 8, we can see that better results are obtained as compared to SOA for pitch angles. However, CNNs and KCovGA algorithms were performing better at pose −30${}^{{}^{\circ}}$.

For the remaining three data-sets (AFLW, BU and ICT-3DHPE), the results were previously reported in the literature for MAE values only. For a fair comparison, we also compared our results with SOA for MAE only. The summary of the results for all the three cases is reported and compared with SOA in Table 2, Table 3 and Table 4 for all the three data-sets respectively. From the tables, it is clear that we had better results in the two cases (BU and ICT-3DHPE) and competitive results for the AFLW database.

AFLW is a database that is collected from the internet. All the images in AFLW are real-world images which are obtained in un-constrained conditions. Importantly, the quality of the images in most of the cases is very poor. Due to this reason, our proposed MSF-CRFs model was not producing promising segmentation results. As a result, we had poor performance as can be seen in the Table 2.

The BU and ICT-3DHPE data-sets are also collected in the real-wold conditions. However, in these cases, the quality of the images is much better. We had better results for both the BU and ICT-3DHPE data-sets, as can be seen in the Table 3 and Table 4.

From the head pose estimation results, it is clear that we had better results in most of the cases, even considering recently proposed CNNs based methods. Through this comparison, we are not disparaging deep learning based methods—rather we believe we need better understanding of the deep learning based methods and their implementation to various tasks.

### 6.3. Age Classification

We reported our age and gender recognition results with term the Classification Rate (CR). We use Adience data-set for age classification. The Adience data-set has eight age categories. We manually labeled 10 images from each age category. A total of 80 images were used to build the MSF-CRFs model for age test. The MSF-CRFs model was used to create segmented images and probability maps. After generating probability maps for all images and all classes, 10-fold cross validation experiments were performed on the remaining images (excluding 80 images which were previously used to build MSF-CRFs model).

For age classification we tried all combination of facial features, as in head pose estimation (excluding background). We noticed that every face part contributed to the age classification. The results reported with HAG-MSF-CRFs and its comparison with SOA are shown in Table 5. From the Table 5, It is clear that we had better results for Adience data-set. Interestingly, for age classification we obtained better results as compared to previous results by a big margin.

We created Ground truth masks through a commercial image editing software. We did this labeling without any automatic segmentation tool. Such kind of labeling has two main drawbacks. Firstly, this labeling highly depends on subjective perception of a single subject involved in this labeling process. Hence it is very difficult to provide an accurate label to all pixels in an image—particularly on the boundary region of the different face parts. For example, differentiating the nose region from the skin and drawing a boundary between the two is very difficult. Secondly, creating manually labeled images is very time consuming and tedious work. Due to this reason, our age part is limited to age classification only. We did not perform tests on the regression part of the age task. For that case, we would need a large number of manually labeled face images for each age number.

### 6.4. Gender Recognition

We performed gender recognition tests with three data-sets, which included Adience, LFW and FERET. The CR values for all three data-sets are shown in Table 6. We also compared our reported results with SOA methods in Table 6.

As in head pose estimation, the possible combinations for all facial features were tried. We obtained the best results with skin, hair, eyes, and nose. After localizing face parts, each image was re-scaled to a height 256 and width was varied accordingly. We manually labeled 30 images from each gender and each data-set. A total of 60 images were used to train an MSF-CRFs (gender) model for each database individually. We performed no cross tests, same database images were used to train an MSF-CRFs model and then some other images of the same data-set were used to evaluate the model.

A fair and exact comparison is very hard to achieve, as different authors use different image settings and different validation protocols. For evaluation of gender recognition, we performed 10-fold cross validation experiments. We manually labeled 60 images, performed 10-fold cross validation experiments, while excluding 60 images which were previously used to build MSF-CRFs model for gender.

Gender classification results with proposed HAG-MSF-CRFs and its comparison with SOA are reported in Table 6. In general, classification accuracy was better than previously reported results. Again, we had poor results as compared to other results for LFW data-set.

As a whole, performance of the newly proposed HAG-MSF-CRFs was very interesting. We introduced a new idea of face image analysis which is using pixel level labeling information for a face image. In a nutshell, we derived an important observation from the reported results *"a strong correlation exists between face parts segmentation and its pose, age and gender. An accurate face segmentation leads to exact head pose, age and gender recognition and vice versa."*

## 7. Conclusions

In this paper we propose an end-to-end semantic face segmentation algorithm (MSF-CRFs) which tries to solve the challenging problems of head pose, age, and gender recognition. The segmentation model is built using the idea of CRFs between various face parts. Three kinds of features are extracted to build the segmentation model. The MSF-CRFs model classify each pixel in the face image to one of the six classes (hair, eyes, skin, nose, mouth, and background). A probabilistic classification strategy is used to generate probability maps for each face class. Random Decision Forest classifier is trained for each task (head pose, age and gender) through different probability maps combination. A large number of experiments are conducted to know which face parts help in head pose, age and gender recognition. Experimental results are validated on six different face data-sets obtaining better or competitive results compared to SOA.

The segmentation results provide sufficient information for different hidden variable in a face image. A route towards different more classification problems in a face image is provided. For example, we are planing to add some more tasks to the framework such as complicated facial expression recognition, ethnicity classification and many more. We are also planing to improve performance of the segmentation part for example using recently introduced CNNs based methods.

## Figures and Tables

**Table 1:** Head pose estimation results and its comparison with SOA on Pointing’04 database.

Method	MAE (Yaw)	Accuracy (Yaw)	MAE (Pitch)	Accuracy (Pitch)
**HAG-MSF-CRFs**	**2.32${}^{{}^{\circ}}$**	**87.7%**	**1.18${}^{{}^{\circ}}$**	**95.0%**
MSF-HPE [14]	3.75${}^{{}^{\circ}}$	77.40%	–	–
MLD [37]	4.24${}^{{}^{\circ}}$	73.30%	6.45${}^{{}^{\circ}}$	86.24%
CNN [60]	5.17${}^{{}^{\circ}}$	69.88%	5.36${}^{{}^{\circ}}$	77.87%
MGD [61]	6.90${}^{{}^{\circ}}$	64.51%	8.00${}^{{}^{\circ}}$	62.72%
kCovGa [62]	6.34${}^{{}^{\circ}}$	–	7.14${}^{{}^{\circ}}$	–
CovGA [62]	7.27${}^{{}^{\circ}}$	–	8.69${}^{{}^{\circ}}$	–

**Table 2:** Head pose estimation results and its comparison with SOA on AFLW database.

Method	Pitch	Yaw	Roll	Average
**QuatNet** [63]	**4.31${}^{{}^{\circ}}$**	**3.93${}^{{}^{\circ}}$**	**2.59${}^{{}^{\circ}}$**	**3.61${}^{{}^{\circ}}$**
HAG-MSF-CRFs	4.89${}^{{}^{\circ}}$	4.25${}^{{}^{\circ}}$	3.20${}^{{}^{\circ}}$	4.11${}^{{}^{\circ}}$
HyperFace [64]	5.33${}^{{}^{\circ}}$	6.24${}^{{}^{\circ}}$	3.29${}^{{}^{\circ}}$	4.96${}^{{}^{\circ}}$
Multi-Loss [65]	5.89${}^{{}^{\circ}}$	6.26${}^{{}^{\circ}}$	3.82${}^{{}^{\circ}}$	5.32${}^{{}^{\circ}}$

**Table 3:** Head pose estimation results and its comparison with SOA on BU database.

Method	Pitch	Yaw	Roll	Average
**HAG-MSF-CRFs**	**2.9${}^{{}^{\circ}}$**	**2.1${}^{{}^{\circ}}$**	**2.2${}^{{}^{\circ}}$**	**2.4${}^{{}^{\circ}}$**
OpenFace2.0 [66]	3.2${}^{{}^{\circ}}$	2.4${}^{{}^{\circ}}$	2.4${}^{{}^{\circ}}$	2.6${}^{{}^{\circ}}$
OpenFace [67]	3.3${}^{{}^{\circ}}$	2.8${}^{{}^{\circ}}$	2.3${}^{{}^{\circ}}$	2.8${}^{{}^{\circ}}$
Chehra [68]	4.6${}^{{}^{\circ}}$	3.8${}^{{}^{\circ}}$	2.8${}^{{}^{\circ}}$	3.8${}^{{}^{\circ}}$
CLM [6]	3.5${}^{{}^{\circ}}$	3${}^{{}^{\circ}}$	2.3${}^{{}^{\circ}}$	2.9${}^{{}^{\circ}}$
FLPD [69]	5.3${}^{{}^{\circ}}$	4.9${}^{{}^{\circ}}$	3.1${}^{{}^{\circ}}$	4.4${}^{{}^{\circ}}$

**Table 4:** Head pose estimation results and its comparison with SOA on ICT-3DHP database.

Method	Pitch	Yaw	Roll	Average
**HAG-MSF-CRFs**	**3.2${}^{{}^{\circ}}$**	**2.6${}^{{}^{\circ}}$**	**2.7${}^{{}^{\circ}}$**	**3.0${}^{{}^{\circ}}$**
OpenFace2.0 [66]	3.5${}^{{}^{\circ}}$	3.1${}^{{}^{\circ}}$	3.1${}^{{}^{\circ}}$	3.2${}^{{}^{\circ}}$
OpenFace [67]	3.6${}^{{}^{\circ}}$	3.6${}^{{}^{\circ}}$	3.6${}^{{}^{\circ}}$	3.6${}^{{}^{\circ}}$
CLM [6]	4.2${}^{{}^{\circ}}$	4.8${}^{{}^{\circ}}$	4.5${}^{{}^{\circ}}$	4.5${}^{{}^{\circ}}$
Reg. Forest [70]	9.4${}^{{}^{\circ}}$	7.2${}^{{}^{\circ}}$	7.5${}^{{}^{\circ}}$	8.0${}^{{}^{\circ}}$
Chehra [68]	14.7${}^{{}^{\circ}}$	13.9${}^{{}^{\circ}}$	10.3${}^{{}^{\circ}}$	13.0${}^{{}^{\circ}}$

**Table 5:** Comparative experiments on age classification using Adience databas.

Method	Database	CR (%)
**HAG-MSF-CRFs**	**Adience**	**66.5**
Dehghan et al. [71]	Adience	61.3 ± 3.7
Hou et al. [72]	Adience	61.1 ± NR
CNNs-EML [41]	Adience	45.1 ± 2.6
Hassner et al. [73]	Adience	50.7 ± 5.1
CNN-ELM [41]	Adience	95.00

**Table 6:** Comparative experiments on gender recognition using Adience, LFW and FERET data-sets.

Method	Database	CR (%)
**HAG-MSF-CRFs**	**Adience**	**89.7**
Levi et al. [74]	Adience	86.8
Lapuschkin et al. [75]	Adience	85.9
CNNs-EML [41]	Adience	**77.8**
Hassner et al. [73]	Adience	79.3
**Van et al.** [76]	**LFW**	**94.4**
HyperFace [64]	LFW	94.0
LNets+ANet [77]	LFW	94.0
HAG-MSF-CRFs	LFW	93.9
Moeini et al. [78]	LFW	93.6
PANDA-1 [79]	LFW	92.0
ANet [40]	LFW	91.0
Rai and Khanna [80]	LFW	89.1
**HAG-MSF-CRFs**	**FERET**	**100**
Moeini et al. [78]	FERET	99.5
Tapia and Perez [81]	FERET	99.1
Rai and Khanna [80]	FERET	98.4
Afifi and Abdelrahman [82]	FERET	99.4
A priori-driven PCA [83]	FERET	84.0
